# Treatment and prevention of lipoprotein(a)-mediated cardiovascular disease: the emerging potential of RNA interference therapeutics

**DOI:** 10.1093/cvr/cvab100

**Published:** 2021-03-26

**Authors:** Daniel I Swerdlow, David A Rider, Arash Yavari, Marie Wikström Lindholm, Giles V Campion, Steven E Nissen

**Affiliations:** Silence Therapeutics plc, 72 Hammersmith Road, London W14 8TH, UK; Silence Therapeutics plc, 72 Hammersmith Road, London W14 8TH, UK; Experimental Therapeutics, Radcliffe Department of Medicine, University of Oxford, John Radcliffe Hospital, Headington, Oxford OX3 9DU, UK; Silence Therapeutics plc, 72 Hammersmith Road, London W14 8TH, UK; Silence Therapeutics plc, 72 Hammersmith Road, London W14 8TH, UK; Department of Cardiovascular Medicine, Cleveland Clinic Foundation, 9500 Euclid Avenue, Cleveland, OH 44195, USA

**Keywords:** Lipoprotein(a), Cardiovascular disease, RNA interference, Therapeutics, Valvular disease

## Abstract

Lipid- and lipoprotein-modifying therapies have expanded substantially in the last 25 years, resulting in reduction in the incidence of major adverse cardiovascular events. However, no specific lipoprotein(a) [Lp(a)]-targeting therapy has yet been shown to reduce cardiovascular disease risk. Many epidemiological and genetic studies have demonstrated that Lp(a) is an important genetically determined causal risk factor for coronary heart disease, aortic valve disease, stroke, heart failure, and peripheral vascular disease. Accordingly, the need for specific Lp(a)-lowering therapy has become a major public health priority. Approximately 20% of the global population (1.4 billion people) have elevated levels of Lp(a) associated with higher cardiovascular risk, though the threshold for determining ‘high risk’ is debated. Traditional lifestyle approaches to cardiovascular risk reduction are ineffective at lowering Lp(a). To address a lifelong risk factor unmodifiable by non-pharmacological means, Lp(a)-lowering therapy needs to be safe, highly effective, and tolerable for a patient population who will likely require several decades of treatment. N-acetylgalactosamine-conjugated gene silencing therapeutics, such as small interfering RNA (siRNA) and antisense oligonucleotide targeting *LPA*, are ideally suited for this application, offering a highly tissue- and target transcript-specific approach with the potential for safe and durable Lp(a) lowering with as few as three or four doses per year. In this review, we evaluate the causal role of Lp(a) across the cardiovascular disease spectrum, examine the role of established lipid-modifying therapies in lowering Lp(a), and focus on the anticipated role for siRNA therapeutics in treating and preventing Lp(a)-related disease.

## Graphical Abstract

**Figure:**
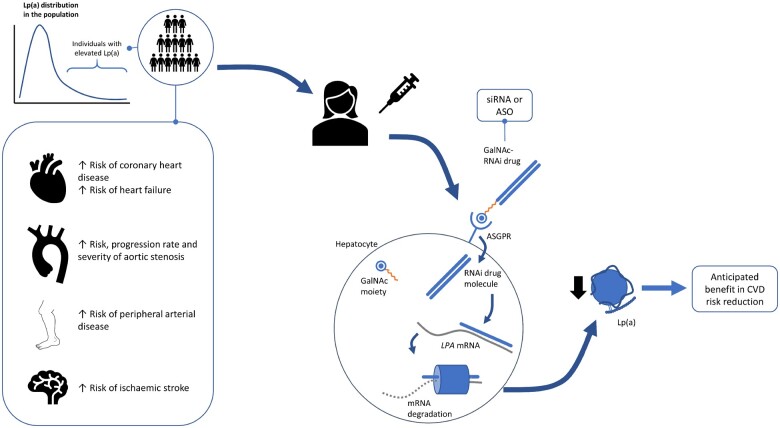

## 1. Overview: the current status of Lp(a) in cardiovascular disease

### 1.1 Lipoprotein(a)—emergence as a risk factor

Lipoprotein(a) [Lp(a)] was first identified and reported in 1963^1^ with evidence supporting its role as a cardiovascular (CV) risk factor accumulating steadily over the last five decades. However, the most informative insights into its causal role in risk for CV disease (CVD) have been generated during the last decade. Much of these data have been generated from large population-based observational studies^2^^,^^3^ and from *post hoc* analyses of trials studying other lipid-lowering agents.^4^^,^^5^ These analyses demonstrate a log-linear relationship between Lp(a) and risk of myocardial infarction (MI), stroke,^3^ and peripheral arterial disease (PAD).^6^ Although effective interventions are now available for many other lipid-related CV risk factors, elevated Lp(a) remains a largely untreatable dyslipidaemia.

Following the introduction of HMG-CoA reductase inhibitors (statins) in 1987, lipid-modifying therapies for the management of CVD have focused largely on modification of low- and high-density lipoprotein cholesterol (LDL-C and HDL-C) and triglycerides. Large randomized outcome studies have demonstrated important benefits of statins,^7^ ezetimibe,^8^ and fibrates^9^^,^^10^ for reducing risk of CV events, chiefly through lowering circulating levels of LDL-C. More recently, novel therapeutic targets, such as proprotein convertase subtilisin/kexin type 9 (PCSK9)^11^^,^^12^ and ATP citrate lyase, the target of bempedoic acid, have shown efficacy^13^ in lowering LDL-C. There have also been notable failures, most prominently niacin and cholesteryl ester transfer protein (CETP) inhibitors. While these approaches to modulation of HDL-C and other lipid fractions have not proved successful, HDL-C remains a potentially valuable route to reduction of CVD risk.^14^ This arsenal of lipid-modifying therapies offers excellent reduction in risk of CVD events through reduction in LDL-C and, possibly, triglycerides. However, existing therapies provide only modest reductions in Lp(a). Consequently, for many patients who have either effective control of other risk factors and persistently raised Lp(a), or for whom Lp(a) is their sole risk factor, a large portion of their risk remains unaddressed.

## 2. The Lp(a) particle and its role in health and disease

The biochemistry and pathophysiological properties of Lp(a) have been described in detail previously;^15^^,^^16^ here, we review the key features relevant to Lp(a)-lowering therapy. The Lp(a) particle is an apolipoprotein B (apoB)-containing lipoprotein similar to LDL, but with apolipoprotein(a) [apo(a)] covalently bound to apoB-100 on the surface of the particle.^17^ Apo(a) itself is a protein encoded by the *LPA* gene (chromosome 6q25.3) that bears substantial homology with plasminogen (*PLG*, chromosome 6q26). Lp(a) and PLG are characterized by kringle motifs, which are protein domains that fold into loops stabilized by three disulphide linkages. Kringle motifs I (KI)–V (KV) are present in PLG, whereas only KIV and KV are found in Lp(a).^17^ In contrast to kringle IV of PLG, in apo(a) KIV is present in 10 subtypes (KIV 1–10) with a single copy of each; the exception is KIV2, which can vary in copy number from 2 to more than 40 identical copies underlying the substantial size heterogeneity of apo(a) in the population. Thus, Lp(a) exists as multiple isoforms, the size of which is determined by the number of repeated KIV2 motifs. The number of repeats is genetically encoded by well-characterized variants at the *LPA* locus.^18^ Both alleles of these variants contribute to the kringle repeat number and serum levels of Lp(a) in individual patients. The Lp(a) isoform size has two important clinical implications. First, smaller isoforms (i.e. those with fewer KIV repeats) have greater atherogenic potential than the same circulating concentration of Lp(a) particles containing larger proteins with more KIV repeats.^19^ Second, the number of KIV repeats can lead mass-based assays (i.e. those reporting circulating concentration in mg/dL) to underestimate the true Lp(a) concentration in the presence of small Lp(a) isoforms. For this reason, the use of molar concentration-based assays (i.e. particle concentration-based reporting in nmol/L) has been increasingly advocated since these account adequately for isoform size, mitigating against underestimating levels of small Lp(a) isoforms or overestimating of large Lp(a) isoforms.^20^^,^^21^

Lp(a) has physiological and pathological roles. Its role in health is diverse and includes modulation of coagulation through its interactions with PLG and the fibrinolytic system, immune cell interaction with the vascular endothelium, and proliferation of vascular smooth muscle and adhesion molecules.^22^ Lp(a) is a major carrier of oxidized phospholipid (OxPL) in human plasma.^23^ OxPLs are mediators of potent proatherogenic and pro-inflammatory effects and are thought to account for a significant proportion of the CV risk attributable to Lp(a).^24^  *LPA* variants [genetically determining increased plasma Lp(a) levels and apo(a) isoform size] strongly associate with OxPL–apoB concentration.^19^ In disease, Lp(a) also has several roles, of which three are most prominent: (i) Lp(a) crosses the vascular endothelium and contributes to the formation and progression of atherosclerotic plaques.^25^ (ii) In the event of atheromatous plaque rupture, the prothrombotic properties of Lp(a)—including inhibition of plasminogen activation to inhibit fibrinolysis—potentiate thrombus formation and contribute to the onset of MI or ischaemic stroke. (iii) Lp(a) crosses the intima of the aortic valve leaflets, promoting inflammation, calcification, and, eventually, aortic valve stenosis.^25^ The role of Lp(a) in specific manifestations of CVD is discussed in more detail below.

## 3. Lp(a) epidemiology and relationship with CVD risk

### 3.1 The pathological relevance of Lp(a) for CVD

The profile of Lp(a) as a CV risk factor has several important differences compared with more established risk factors. Unlike LDL-C, blood pressure, and body mass index (BMI), the population distribution of circulating Lp(a) concentration is positively skewed rather than normally distributed. The majority of the population (∼80%) have concentrations of Lp(a) below ∼50 mg/dL (∼125 nmol/L),^25^ including a group of individuals with very low or undetectable circulating Lp(a).^26^ Importantly, those individuals are not known to experience any adverse consequences of their low Lp(a) thereby providing reassurance regarding the safety of large reductions using therapeutic interventions seeking to recapitulate this pharmacologically.^27^ The population distribution of Lp(a) also differs by ancestral group. People of African ancestry have, on average, higher circulating Lp(a) than people of European or Asian ancestry,^28^^,^^29^ raising important considerations for risk prediction and modification given the inter-ancestral variation of other risk factors, such as hypertension and dysglycaemia.

A person’s genotype regulates Lp(a) kringle number and isoform size; kringle IV repeats are the most important, but not the sole, determinant of serum concentrations, with *LPA* genotype accounting for >90% of circulating Lp(a) variance.^30^ Circulating Lp(a) concentration is determined at the time of conception and, with few exceptions, such as the influence of renal dysfunction and perimenopausal hormonal changes,^31^^,^^32^ varies little during their lifetime. In contrast, the development of other risk factors, such as LDL-C, blood pressure, and BMI, is predominantly a feature of middle age and beyond.^33^ These conventional risk factors often develop with aging and are subject to the influence of behaviour, diet, and concomitant disease. In the case of Lp(a), raised levels are detectable and fixed much earlier in life^34^^,^^35^ and the circulating concentration remains relatively stable as a person ages. The adverse effects of raised Lp(a) on disease risk are therefore present from an earlier stage in life than many other risk factors. Thus, earlier intervention to lower Lp(a) before the accumulation of additional risk later in life may offer greater benefit for prevention. Evidence suggests that the relationship of elevated Lp(a) with risk of recurrent CVD events may not be equivalent to that with risk of first CVD events.^36–38^ However, as discussed below, clinical trials are underway to provide deeper insights into those relationships.

Our understanding of the relevance of Lp(a) in many manifestations of CVD comes from observational studies, population genetic studies, and a few interventional trials. Observational studies, particularly those with longitudinal information on incident disease provide important epidemiological insights about the relationship between higher Lp(a) and higher risk of CV events, but do not permit causal inference. Genetic studies that evaluate associations of genetic variants at the *LPA* locus with Lp(a) concentrations enable causal inference using the Mendelian randomization paradigm^39^ and have been instrumental in elucidating the potential for Lp(a) lowering as a promising therapeutic strategy in CVD.^40–42^ Finally, randomized trials of lipid-modifying agents, such as statins and PCSK9 inhibitors with some effect on Lp(a) have offered some limited information on the value of Lp(a) lowering, although interpretation of these data is limited by the lack of specificity of the drugs tested for an effect on Lp(a).^43–45^

### 3.2 Coronary heart disease

The relationship between raised Lp(a) and risk of coronary heart disease (CHD) events is strongly supported by multiple observational studies. A large meta-analysis of longitudinal observational studies reported a risk ratio of 1.16 [95% confidence interval (95% CI) 1.11–1.22] for CHD for each 1 standard deviation higher Lp(a), which was equivalent to 3.5-fold higher Lp(a) in the population studied.^3^ This strong relationship was confirmed in a large Danish cohort with participants with Lp(a)≥20 mg/dL (∼300 nmol/L)—representing the 95th centile—demonstrating a 20% higher 10-year absolute risk of MI in female patients and 35% higher in males over 60 years in age who smoked and had hypertension.^2^

Genetic studies have supported a causal association underlying these observational estimates of the relationship between Lp(a) and CHD. Although circulating Lp(a) levels may differ markedly between ancestral groups,^46^ the Lp(a)–CHD risk association is observed in multiple populations.^29^ Genome-wide association studies (GWASs) have identified the *LPA* locus as strongly associated with risk of coronary disease. Two variants (rs10455872 and rs3798220) are both highly associated with increased plasma Lp(a), reduced *LPA* copy number (i.e. reduced KIV2 repeats), and smaller Lp(a) lipoprotein size—associated with an odds ratio for coronary artery disease of 1.5 for one variant and 2.57 for two or more variants.^40^ Mendelian randomization studies show a genetically determined doubling of Lp(a) plasma levels results in a 22% increased risk of MI during a maximum of 16 years of follow-up, consistent with a causal relationship between lifelong elevation in Lp(a) and MI.^41^ Two Mendelian randomization studies have sought to quantify the likely magnitude of Lp(a) reduction in a clinical trial equivalent to lower LDL-C by 1 mmol/L (38.67 mg/dL) with respect to CHD risk reduction.^47^^,^^48^ The estimates of the equivalent Lp(a) reduction ranged from a 65.7 mg/dL affording ∼22% relative risk reduction,^48^ to 101.5 mg/dL affording ∼24% CHD relative risk reduction.^47^ These estimates rest on the assumption that lifelong genetically determined circulating LDL-C and Lp(a) levels have approximately equivalent effects on CHD risks. While this assumption is supported by epidemiological data, it suggests that even large Lp(a) reductions in Lp(a) may confer only modest CHD risk reduction. However, the data used to generate these estimates came from general population samples with relatively low median baseline Lp(a) (range: 11–43.3 mg/dL). The potential for benefit in patients at the higher end of the skewed Lp(a) distribution (i.e. >50 mg/dL) may be greater. Furthermore, the findings of Mendelian randomization analyses reflect lifelong effects of genetic variants, which may differ in magnitude from the effects of drug treatment which is, in general, of relatively shorter duration and greater potency.

### 3.3 Aortic valve stenosis

Lp(a) shows a strong positive relationship with aortic stenosis (AS) in observational and genetic studies.^49^^,^^50^ Elevated Lp(a) and OxPL-apoB are independently associated with more rapid progression of stenosis in patients with mild-moderate AS, with patients in the top tertile of Lp(a) or OxPL-apoB exhibiting increased valvular calcification activity—measured by ^18^F-NaF PET/CT—compared with those in the lower tertiles, and a higher risk of aortic valve replacement (AVR) or cardiac death.^49^^,^^51^ Notably, the relationship between Lp(a) and OxPL levels with rate of AS progression appears to be linear rather than a threshold association.^51^ In a *post hoc*, exploratory analysis of the FOURIER trial of the PCSK9 inhibitor evolocumab, higher Lp(a), but not higher LDL-C, was associated with greater likelihood of new AS diagnoses or AS worsening over ∼2 years of follow-up.^52^ Genetic data also implicate Lp(a) as a key mediator in the pathogenesis and development of AS. A GWAS of aortic valve calcium burden detected by CT imaging identified a variant in *LPA* (rs10455872) ^40^ that was associated with approximately a two-fold increase in odds of aortic valve calcification. The same variant was significantly associated with incident AS.^53^ Using a Mendelian randomization approach, the same study identified a relationship between genetically determined higher Lp(a) levels and higher likelihood of the presence of aortic valve calcification. The association between this and other *LPA* variants, serum Lp(a) levels and AS has been replicated by other studies,^54^ including those employing a prospective Mendelian randomization design.^55^ Recent Mendelian randomization analyses have suggested a causal role for Lp(a) in other lesions of the aortic and mitral valves.^42^

### 3.4 Other manifestations of CVD

Beyond the two major disease associations described above, Lp(a) plays an important role in the risk of several other CVDs. Higher circulating Lp(a) levels associate with higher risk of ischaemic stroke in cohorts across ancestral groups.^56–58^ Notably, the association with ischaemic stroke was observed in individuals without atrial fibrillation (AF) but not in those with AF,^59^ which may suggest Lp(a) promotes an atheroembolic mechanism of cerebral infarction rather than a cardioembolic aetiology. Genetic studies also support a causal relationship between Lp(a) levels and ischaemic stroke risk.^60^ PAD associates strongly with higher Lp(a) levels, as does greater likelihood of peripheral revascularization in people with established PAD, suggesting Lp(a) has a role in not only the onset of disease, but also its progression.^6^^,^^53^^,^^61^^,^^62^ The role for Lp(a) in the epidemiological relationship with PAD is also supported by genetic studies. In a large GWAS of PAD risk, a single nucleotide polymorphism (SNP) in *LPA* was the variant most strongly associated with PAD risk.^63^ Heart failure is a disease phenotype closely related to CHD and AS and displays an observational relationship with Lp(a).^64^^,^^65^ Large-scale genetic studies support this relationship^66^ but the association between *LPA* genotype and heart failure is attenuated after adjustment for accompanying CHD, suggesting that the role of Lp(a) in heart failure is largely mediated through coronary atherothrombotic disease. *LPA* genotype and elevated Lp(a) were associated with younger age at death of participants’ parents in a large GWAS study, with the same genetic variant associated with higher Lp(a) levels and higher CHD risk.^67^

Taken together, the diseases in which Lp(a) plays an important causal role make a compelling case for the potential benefit to individual and population health of specific, safe, and effective Lp(a)-lowering therapy.

### 3.5 Lp(a) and familial hypercholesterolaemia

Familial hypercholesterolaemia (FH) is a collection of disorders characterized by very high circulating LDL-C levels largely caused by mutations at the *LDLR, PCSK9*, and *APOB* genetic loci. While high LDL-C in many patients with FH can be managed effectively with pharmacological and lifestyle measures, raised Lp(a) can confer a sizable burden of additional risk.^68^ In patients with heterozygous FH, median Lp(a) has been reported to be up to almost two-fold higher than the general population,^68^ although published findings on this subject vary,^22^^,^^69^^,^^70^ and raised Lp(a) is an independent predictor of CVD risk in patients with FH.^70^ These findings have led to recommendations to incorporate Lp(a) measurement in the systematic cascade screening of patients with molecularly defined FH, identifying 1 new case for every 2.4 screened.^71^ Patients with FH therefore carry two potent, genetically determined drivers of CVD risk,^72^ including risk of both CHD^73^ and AS.^74^ Patients with FH are therefore a group who may benefit from novel Lp(a)-lowering therapies.

## 4. Current options for management of Lp(a)-mediated CV risk

As summarized in *Table 1*, the effects of established lipid-modifying agents on Lp(a) are both modest and variable, with only trials of the monoclonal antibody PCSK9 inhibitors reporting a potentially clinically meaningful reduction in Lp(a). While PSCK9 inhibitors demonstrate some Lp(a)-lowering effect, the magnitude of the effect is likely insufficient to adequately reduce Lp(a)-mediated CVD risk in the population.^47^^,^^48^ The CETP inhibitors anacetrapib and evacetrapib reduced Lp(a) by 25% and 40%, respectively.^75^^,^^76^ These drugs did not demonstrate an overall benefit for CVD risk reduction but, importantly, were evaluated in trials that did not enrol patients on the basis of elevated Lp(a).

**Table 1:** Effects of established lipid-modifying agents on Lp(a)

Drug or drug class	Effect on Lp(a)
Statins	Substantial heterogeneity between statin drugs in a meta-analysis of RCTs. Effects ranged from 13% reduction (95% CI 10–15%) for atorvastatin in the CARDS study^82^ to 15% increase (95% CI 13–17%) for simvastatin in the 4S study.^131^ No overall effect when data meta-analysed leading to uncertainty of the effect of statins.^43^
Ezetimibe	No significant effect in a meta-analysis of RCTs.^44^
Niacin	Reduced by 22.9% (95% CI 18.5–22.9%) in a meta-analysis of RCTs.^132^ Effect was not dose-dependent.
Fibrates	No significant effect in a meta-analysis of RCTs.^133^
Bempedoic acid	No significant effect on Lp(a) in phase 2 study.^134^
PCSK9 inhibitor monoclonal antibodies	Median Lp(a) reduction with evolocumab was 26.9% [interquartile range (IQR) 6.2–46.7%) in the FOURIER study.^5^ Median reduction with alirocumab was 25.6% (IQR 7.2–42.7%) in pooled phase 3 trial data.^135^ This was confirmed in a pooled analysis.^45^
Inclisiran	18.6% reduction from baseline in the phase 3 ORION-11 study.^136^
Mipomersen	Median Lp(a) reduction in pooled phase 3 trials was 26.4% (IQR 5.4–42.8%).^137^
CETP inhibitors	Evacetrapib reduced Lp(a) by up to 40% in a phase 2 study.^76^ Anacetrapib reduced Lp(a) by 34.1% in a small phase 2 study.^138^

Lipoprotein apheresis offers an effective means of lowering apoB-containing lipoproteins, including LDL and Lp(a).^77^ Apheresis is an intensive treatment modality that requires patients to attend a specialist centre at 1–2 weekly intervals for treatment sessions lasting between 90 min and 4 h. Apheresis is successful in reducing both Lp(a) and LDL-C from baseline by ∼70% and 65%, respectively, although the reduction in Lp(a) follows a saw-tooth pattern with modest time-averaged reductions.^78^ Apheresis is recommended by clinical guidelines in only a small number of countries including Germany. In general, apheresis is offered to patients in the context of high Lp(a) (e.g. >60 mg/dL in Germany) with evidence of progressive CVD or recurrent CVD events despite optimal management. Apheresis has additional benefits beyond lipoprotein reduction, including lowering plasma viscosity^79^ and removal of pro-inflammatory mediators.^80^ While definitive data from randomized trials of apheresis in CVD event prevention are not available, prospective non-randomized studies, such as Pro(a)LiFe, which included 170 patients treated with apheresis, have suggested substantial reductions in event rates.^78^ In addition to the inconvenience for patients, apheresis carries some risk of adverse events. The majority of these are related to vascular access, and transient haemodynamic effects of extracorporeal circulation, such as dizziness.^78^^,^^79^

In addition to the treatment effects on Lp(a) reported in randomized trials of lipid-lowering drugs, these trials have shown interactions between baseline circulating Lp(a) levels and the effects of treatment on CV risk. In the JUPITER study, Lp(a) levels were associated with residual risk of CVD in participants treated with rosuvastatin, independently of LDL-C lowering.^4^ The ODYSSEY-OUTCOMES study reported that the effect of alirocumab mediated by Lp(a) reduction reduced major adverse cardiovascular events following an acute coronary syndrome independent of the effect mediated by LDL-C lowering.^81^ While encouraging, these *post hoc* findings from a trial of a drug principally targeting LDL-C will benefit from dedicated investigation in trials of specific Lp(a)-lowering agents. Pharmacogenetic analyses in statin RCTs have demonstrated an effect of genotype at the *LPA* locus (and for the rs10455872 variant in particular). In the CARDS and PROSPER studies, Lp(a)-raising alleles of the rs10455872 SNP were associated with a smaller reduction of LDL-C on atorvastatin treatment; in CARDS, however, there was no evidence of interaction between genotype and CVD end points.^82^ A similar finding was observed with simvastatin treatment in the Heart Protection Study,^83^ with rosuvastatin in the JUPITER study^84^ and in a population-based observational study in Scotland.^85^ Importantly, these observations may be confounded by the methods used to measure LDL-C, the results of which likely incorporate the cholesterol content of both LDL and Lp(a) particles (constituting ∼30–45% of the latter) and therefore do not entirely reflect the specific influence of *LPA* genotype on LDL-C.^83^ In a population-based GWAS of CHD events in individuals receiving statin treatment, the *LPA* rs10455872 SNP was the variant most significantly associated with higher CHD risk in statin-treated participants, an association that was independent of statin-induced LDL-C reduction.^86^

Risk for AS merits particular mention given the important role Lp(a) appears to play in the development and progression of this valve disorder. No pharmacotherapy is currently available to slow or reverse the progression of AS. Currently, the only option available for patients with severe symptomatic AS is surgical or transcatheter aortic valve replacement.^87^ In the stages of AS before disease progression necessitates AVR, the management of AS comprises longitudinal surveillance of valve function, management of left ventricular dysfunction and modification of CHD risk which often coexists with AS.^88^ RCTs investigating the effects of statin therapy in AS have not demonstrated improvements in aortic valve pressure gradient, jet velocity or valve area, or progression to valve replacement.^89^

Effective management of a lifelong risk factor, such as Lp(a) requires an intervention that is safe for long-term use, effective, and acceptable to the patients who will likely require many years of treatment. For existing oral lipid-modifying therapies administered daily, treatment adherence and persistence can be challenging for many patients. Available observational data suggest that lower treatment adherence and persistence has a strong association with higher risk of CV events.^90–92^ Monoclonal antibody PCSK9 inhibitors can potentially address this challenge through longer dosing intervals of 2–4 weeks.^93^^,^^94^ Long-term drug therapy must also take account of the risk of adverse effects. For statins, the risk of muscle pain and weakness^95^ and new-onset type 2 diabetes^96^ remains a concern to many patients and prescribers. The ideal specific Lp(a)-lowering therapy would have as benign a safety profile as possible and its administration would place the minimum burden on the patient. A major factor in determining that burden is the frequency of administration. Lp(a)-lowering therapy with a RNA interference (RNAi) agent [e.g. small interfering RNA (siRNA)] offers an opportunity for a particularly durable and effective therapy.

## 5. Current clinical guidance on management of raised Lp(a)

Recent revisions of clinical guidelines for CVD risk management and lipid modification in Europe, the USA, Canada, and the UK have addressed the issue of Lp(a) as a risk factor. Notably, the focus of these guidelines is chiefly atherosclerotic and atherothrombotic disease, while aortic valve disease is not directly addressed. The joint American Heart Association/American College of Cardiology Task Force guidelines in 2018 identify circulating Lp(a) over 50 mg/dL (124 nmol/L) as a ‘risk enhancer’ for atherosclerotic CVD (ASCVD), which should be measured particularly if a patient has a family history of premature ASCVD.^97^ The Canadian Cardiovascular Society 2016 guidelines take a similar position, noting that elevated Lp(a) may be of particular value for risk modification in younger patients for whom Lp(a) represents an important lifetime risk factor but might not meet standard risk criteria for treatment.^98^ The Japanese Atherosclerosis Society 2017 guidelines for ASCVD prevention acknowledge Lp(a) as a CVD risk factor but do not include specific recommendations for Lp(a) measurement or the management of risk in people with raised Lp(a).^99^ The European Society of Cardiology (ESC)/European Atherosclerosis Society (EAS) 2019 guidelines acknowledge the promise of RNA-based therapeutics for Lp(a)-lowering and recommend that a single measurement of Lp(a) may help to identify people with very high inherited levels who may have a substantial lifetime risk of ASCVD. A high Lp(a) plasma level may also be helpful in further risk stratification of patients at high risk of ASCVD, in patients with a family history of premature CVD, and to determine treatment strategies in people whose estimated risk falls on the border of risk categories.^100^ Since Lp(a) remains stable throughout adulthood, a single measurement is adequate for screening and therefore the burden on patients and health services of undertaking screening is minimal. Heart UK, a charity supporting patients with hyperlipidaemia in the UK, released a position statement on Lp(a) in 2019.^101^ The statement is broadly in line with the recommendations of the ESC/EAS guidelines and also advocates measurement of Lp(a) in patients with calcific AS and those with equivocal but <15% 10-year risk of CVD events. Further, Heart UK recommend overall reduction of CVD risk including non-HDL-C lowering and consideration of apheresis for the management of patients with elevated Lp(a) levels. The National Lipid Association (NLA) in the USA released a similar position paper in 2019.^21^ The NLA statement makes the important observation that measurement methods for Lp(a) are not yet standardized and that more evidence is needed to support Lp(a) thresholds for determining higher CVD risk in subpopulations defined by age, sex, ancestry, and comorbidity.

## 6. RNAi as an emerging treatment modality for lowering Lp(a)

RNAi is an emerging treatment modality with the potential to address Lp(a)-mediated disease risk. Several RNAi drugs, including siRNAs and antisense oligonucleotides (ASOs), against other therapeutic targets have demonstrated effectiveness in clinical studies and a few have received regulatory approval, such as the two Alnylam siRNA therapeutic agents Onpattro (patisiran; targeting hereditary ATTR amyloidosis, formulated as lipid nanoparticles) and Givlaari (givosiran; targeting acute hepatic porphyria, a GalNAc-conjugated siRNA, see below).

### 6.1 RNAi therapies: mechanism of action and utility in lipid-modifying therapy

Oligonucleotide therapeutics (OT) is a term that by now covers a broad range of modalities, most of which are synthetic oligonucleotides containing sequences of native and modified RNA and DNA nucleosides with phosphorothioate (PS) or modified internucleotide linkages. RNAi drugs, a subset of OT, exploit natural mechanisms for regulating gene expression in human cells.^102^^,^^103^ Unlike gene therapy (e.g. Luxturna for inherited retinal dystrophy^104^) which permanently modifies the patient’s genome, RNAi results in temporary and reversible down-regulation of gene expression. ASOs are single-stranded sequences of native and modified nucleotide molecules that bind to their target complementary RNA sequence via Watson–Crick base pairing. This binding leads to RNA degradation through RNase H enzyme activity, resulting in reduced protein production. Early generation ASOs were beset by challenges to their safety and effectiveness, including susceptibility to degradation by nucleases, poor tissue specific delivery, and a narrow therapeutic range due to class effect toxicities. Some of these challenges have been successfully overcome through chemical modifications to the oligonucleotide molecules and several ASO drugs have been successful in clinical trials. These include mipomersen^105^ for treatment of FH, volanesorsen for treatment of familial chylomicronaemia syndrome^106^ and notably an Lp(a)-lowering ASO, AKCEA-APO(a)-_LRx_.^107^

**Figure 1:**
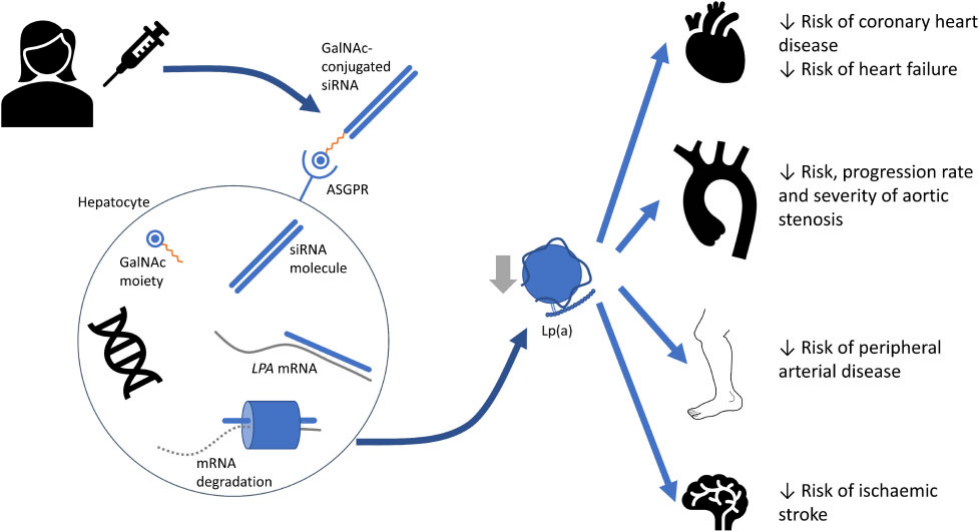
Mechanism of action of Lp(a)-lowering siRNA therapy, and potential clinical benefits. GalNAc–siRNA drugs lower circulating Lp(a) concentrations via the mechanisms described in more detail in *Figure 2*. Evidence from observational and genetic epidemiological studies supports a role for Lp(a) lowering as a means of reducing the risk of several types of cardiovascular disease, including atherosclerotic in the coronary and peripheral circulations, valvular disease and heart failure. siRNA, small interfering RNA; ASGPR, asialoglycoprotein receptor; LPA, apolipoprotein(a) gene; Lp(a), lipoprotein(a).

**Figure 2:**
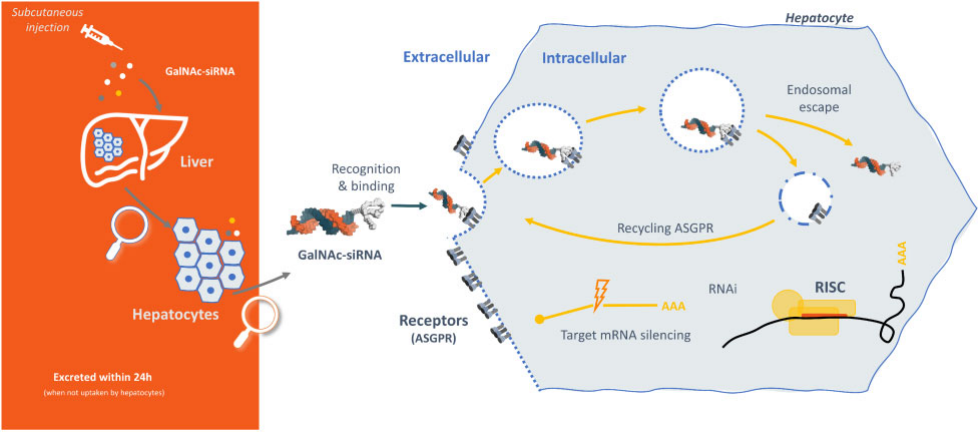
A detailed overview of GalNAc–siRNA mechanism of action in the hepatocyte. A GalNAc–siRNA drug is administered subcutaneously and the GalNAc moiety binds the ASGPR on the hepatocyte cell membrane. The drug conjugate enters the cell via an endosomal mechanism, following which it escapes into the cytosol and the ASGPR recycles to the cell surface. The siRNA sense and antisense strands dissociate, and the antisense strand binds its target sequence in the LPA gene mRNA. Binding of the antisense strand induces degradation of *LPA* mRNA, preventing its translation into apo(a) and thereby reducing the synthesis of Lp(a). GalNAc, N-acetyl-galactosamine; ASGPR, asialoglycoprotein receptor; RISC, RNA-induced silencing complex; RNAi, RNA interference; AAA, gene transcript.

By contrast, siRNA drugs are double-stranded RNA molecules (*Figures 1 and 2*). Once inside the cell, the two RNA strands dissociate into sense and antisense strands by the action of the Argonaute 2 (AGO2) and the antisense strand gets inserted into, and ready to exert mRNA degrading activity in, RNA-induced silencing complex (RISC). The sense strand degrades and the antisense strand binds to its target mRNA sequence again using Watson–Crick base pairing. This binding induces cleavage of the target mRNA by AGO2 and degradation by exonucleases, resulting in reduced synthesis of its cognate protein. Regardless of whether the RNAi agent is a single-stranded ASO or a double-stranded siRNA, stretches of native RNA and DNA are quickly degraded in the circulation and inside cells, and much effort has been done to improve extra- and intracellular RNAi agent stability with retained or improved activity and safety profile. For siRNA, it has been clearly demonstrated that unless the siRNA is delivered encapsulated in a nanoparticle formulation, chemical modification of the siRNA sequence is required to avoid extracellular degradation and excessive renal clearance before the drug reaches its intended cell or tissue.^103^

RNAi molecules need to overcome several barriers in order to be efficacious drugs. First, they need to be stable enough in circulation not to get degraded before they reach their target cell/tissues, second, they need to reach, and be endocytosed into, their target cell/tissue, and third, they need to escape the vesicles of the endo/lysosomal pathway in order to reach their final location—cytosol or nucleus, depending on modality. ASOs, in particular, ASOs stabilized with a PS ‘backbone’, can enter many cells and tissues relatively freely due to cell surface protein binding followed by endocytosis.^108^^,^^109^ In contrast, the larger, double stranded siRNA molecules have a size and charge that strongly reduces non-specific, or unassisted, uptake in target cells and tissues. Different nanoparticle formulations, such as used in Onpattro, will increase siRNA uptake and activity for instance in liver. However, over the last 5–6 years focus has shifted to using direct conjugation of siRNA and ASOs to targeting moieties designed for binding to and internalization through cell-specific receptors The most successful and broadly used conjugation strategy to date, and delivery method pertinent to lipid modification, is conjugation of siRNA or ASO molecule to a cluster of sugars, N-acetylgalactosamines, or GalNAc. GalNAc is a ligand for the asialoglycoprotein receptors, which are found in large concentrations on the surface of hepatocytes with a high degree of cell- type specificity.^110^ Conjugation of an RNAi drug molecule to GalNAc clusters therefore directs the oligonucleotide near-exclusively to the hepatocytes where it can exert its gene silencing activity without incurring risks of doing so in other, unintended cell types. Recent examples of clinically successful GalNAc–siRNAs are givosiran (Givlaari), approved for the treatment of acute hepatic porphyria^111^ and fitusiran for treatment of haemophilia A & B,^112^ with GalNAc-conjugated ASOs also in development.

### 6.2 Safety of GalNAc–RNAi therapy

Given the central role for the liver in lipid metabolism, GalNAc-conjugated siRNAs present a valuable opportunity for treating dyslipidaemia. Inclisiran, a GalNAc-conjugated siRNA inhibitor of PCSK9 is under consideration for approval in several countries following successful phase 3 trials.^113^ Hepatocyte specificity is just one of the features of GalNAc–siRNA drugs that make them attractive candidates for long-term Lp(a)-lowering therapy. The GalNAc–siRNA drugs evaluated in clinical trials to date have demonstrated reassuring safety profiles. Inclisiran demonstrated a modest increase in injection site reactions compared with placebo in two Phase-3 trials that included 1561 participants,^113^ but no excess adverse events in either of these trials or a Phase-2 trial in 482 patients with FH.^114^ A similar safety profile was observed in a Phase-1 trial of givosiran.^111^ Critical to these safety profiles are the chemical modifications to the siRNA molecule. These modifications not only help the drug to avoid premature degradation but also reduce the risk of activating an innate immune response leading to adverse effects related to systemic inflammation.^115^ Risk of off-target binding and interference in the expression of unintended target genes remains a potential concern with RNAi drugs. This risk is mitigated through a careful *in silico* design process during which sequence similarity to off-target genes guide chemical modifications optimized to reduce risk of off-target effects.^116^ These features have overcome many of the difficulties faced by earlier generation RNAi drugs and help establish siRNAs as promising approach suitable for long-term use.

### 6.3 Durability of RNAi treatment effects

A prominent feature of treatment with inclisiran during clinical development is its long duration of action. In the Phase 3 ORION-10 and ORION-11 studies, inclisiran was administered on day 1, day 90, and every 6 months thereafter, resulting in reductions in LDL-C of ∼50% maintained at 17 months of follow-up.^113^ Prolonged treatment durations were also observed with givosiran^111^ and fitusiran,^112^ although the effects were not as durable as observed with inclisiran. Although the pharmacodynamic effect of GalNAc–siRNA drugs persist for weeks or months, these drugs are rapidly cleared from the plasma compartment, chiefly by renal elimination such that circulating levels can be undetectable 24 h after dosing.^117^ The long, or very long, duration of action after single injections of GalNAc-conjugated siRNAs appears to at least in part be explained by chemical modifications leading to increased stability under acidic conditions.^118^ As mentioned above, RNAi molecules must undergo endocytosis followed by release from endo/lysosomal compartments in order to achieve their pharmacological effect. It has been demonstrated that chemical modifications that lead to increased siRNA stability under acidic conditions (such as in late endosomes) are connected with increased duration of action in animal studies.^118^^,^^119^ One possible explanation is that increased siRNA stability in an acidic vesicular compartment leads to formation of an intracellular depot from which a small amount of siRNA is released over time—a hypothesis supported by evidence that chemical modifications leading to a more stable siRNA molecule has no effect on RNAi activity, compared to a less stable version of the same siRNA, once the antisense strand has been inserted in the RISC complex.^118^

This extended duration of action represents an ideal profile for long-term CVD risk reduction. Adherence and persistence are major challenges in optimizing the benefit for CVD risk-modifying drugs. In the case of subcutaneously administered monoclonal antibody inhibitors of PSCK9, dosed at 2- or 4-weekly intervals, adherence has been shown in real-world datasets to be ∼70–80%,^94^^,^^120^ while for statins adherence has been reported as low as 17.8%.^121^ It is anticipated that adherence to long-acting RNAi lipid-modifying drugs, administered less frequently, may be higher than for monoclonal antibodies, resulting in greater clinical benefits. The long duration of action may also provide logistical and financial benefits to healthcare systems derived from the need for less frequent dosing.

## 7. Potential risks of Lp(a)-lowering therapy

The anticipated benefits of reducing Lp(a) are considerable, however, few treatments are without safety concerns. Available clinical studies of Lp(a)-lowering therapies have provided some insights into the potential risks of Lp(a) reduction. However, insufficient person-years of treatment have accumulated to provide a full understanding of the potential adverse effects of lowering Lp(a). Theoretical risks related to the sequence homology with plasminogen exist and thrombosis-related adverse events will require carefully monitored in clinical studies of Lp(a)-lowering agents. Large-scale genetic studies have utility in identifying target-related adverse effects in the same way that they can highlight beneficial therapeutic effects.^122^ A large phenome-wide association study examined the associations between variants at the *LPA* locus and risk of a range of diseases and related biomarkers.^123^ This study confirmed the predicted risk-lowering effects for CHD, AS, heart failure, and PAD. It also identified no associations with potential adverse effects on risk of 28 other disease phenotypes including gastrointestinal, endocrine, neurological, musculoskeletal, respiratory, and neoplastic disease. The analysis also examined relationships between *LPA* genotype and several biomarkers of cardiometabolic disease, demonstrating effects on circulating lipids while detecting no effects on blood pressure, or markers of adiposity or glycaemic control. Interestingly, there was a strong association between Lp(a)-lowering variants and higher estimated glomerular filtration rate, accompanied by a modest association with lower risk of chronic kidney disease. In an Icelandic study that included individuals homozygous for loss-of-function *LPA* mutations and therefore very low circulating Lp(a) concentrations, such variants were associated with a modestly increased risk of type 2 diabetes. While this association merits careful monitoring clinical trials of Lp(a)-lowering drugs, the weight of evidence from observational and genetic epidemiological studies suggests that, as for statins,^124^^,^^125^ the CV benefits Lp(a)-lowering are likely to outweigh any potential risks related to diabetes.

## 8. Emerging Lp(a)-reducing RNAi therapeutics

Novel, specific, RNAi-based Lp(a)-lowering therapies have entered clinical development in recent years. These include pelacarsen [formerly TQJ230 and AKCEA-APO(a)-LRx], an ASO targeting *LPA* originally developed by Akcea Therapeutics now being developed by Novartis under licence; olpasiran (formerly AMG890 and ARC-LPA), a GalNAc-conjugated siRNA originally developed by Arrowhead Pharmaceuticals now being developed by Amgen; and, SLN360, a GalNAc-conjugated siRNA being developed by Silence Therapeutics. The current status of these three molecules is summarized in *Table 2*.

**Table 2:** Lp(a)-lowering RNAi therapeutics in development

Drug	RNAi technology	Industrial sponsor	Stage of development	Route of delivery	Efficacy data to-date	Safety data to-date
AKCEA-APO(a)-L_Rx_	ASO	Novartis—Ionis/Akcea	Phase 3	Sub-cutaneous injection	Dose-dependent Lp(a) lowering in Phase 2 clinical trial in adults with Lp(a) ≥60 mg/dL (≈≥150 nmol/L) and established CVD. Up to 80% reduction (75.1 mg/dL; 187.8 nmol/L) at highest (20 mg weekly) dose in patients with median baseline Lp(a) 224.3 nmol/L (IQR 177.2–286.9).^107^	Adverse events (AEs) more frequent in active arms (90%) than in placebo arm (83%); serious AEs followed a similar pattern (10% active arms; 2% placebo arm). Injection site reactions were the most common AE.^107^
ARC-LPA/AMG890	GalNAc–siRNA	Amgen-Arrowhead	Phase 2	Sub-cutaneous injection	Phase 1 clinical trial showed Lp(a) reduction of over 90% at doses ≥9 mg/kg that persisted 3–6 months.^126^ (https://clinicaltrials.gov/ct2/show/NCT03626662). Phase 2 clinical trial under way (https://clinicaltrials.gov/ct2/show/NCT04270760). In cynomolgus monkeys, 85–90% Lp(a) reduction at 29 days after 3 doses at 3 mg/kg Q1W; >75% reduction persisted at 6 weeks after third dose.^139^	In phase 1 study data, there was no excess of adverse events with AMG890.^126^
SLN360	GalNAc–siRNA	Silence Therapeutics	Phase 1	Sub-cutaneous injection	Phase 1 clinical trial starting in 2021 (https://clinicaltrials.gov/ct2/show/NCT04606602). In cynomolgus monkeys, >95% Lp(a) reduction at 29 days after 3 doses at 3 mg/kg Q1W; >95% reduction persisted at 7 weeks after third dose.^140^	Non-clinical safety studies in Cynomolgus monkey showed no observed adverse effect level was >60-fold greater than pharmacological active dose; <1% of peak liver exposure in tissues outside liver and kidney.^141^

All three molecules display reductions in Lp(a) that are likely to be clinically meaningful. The duration of effect appears to be substantially greater for the siRNA molecules than the ASO—data from the Phase-1 study of olpasiran show Lp(a) reduction of over 90%^126^ compared with reduction of up to 80% with pelacarsen in a Phase-2 study.^107^ Findings with inclisiran in a population of patients similar to those likely to be eligible for Lp(a)-lowering therapy suggest GalNAc-conjugated siRNAs are likely to perform well from a safety perspective. Injection site reactions were the most commonly reported adverse effect of pelacarsen in a Phase-2 study, while these were very rare in the Phase-1 study of olpasiran. If the subcutaneously delivered siRNA drugs, AMG890 and SLN360 prove both safe and effective in the ongoing and planned clinical studies, they stand to offer attractive options for CVD risk modification in patients with raised Lp(a).

## 9. Opportunities for precision medicine in managing raised Lp(a)

The evidence discussed above strongly suggests that reducing circulating Lp(a) is likely to have a beneficial impact on the onset and progression of CVD and risk of CVD events. Emerging drugs have shown promise in achieving safe and effective Lp(a) reduction. For these new medicines to be effective, they must be prescribed for patients most likely to gain the greatest clinical benefit.

Management of Lp(a)-mediated disease presents an opportunity for precision medicine on a large scale. Since circulating Lp(a) levels are largely determined by genotype and only modestly influenced by other modifiable factors, patients eligible for Lp(a)-lowering therapy can be identified in early adulthood. This information may be valuable as a prognostic indicator for CVD. However, like LDL-C, the utility of Lp(a) for risk prediction may differ between first and subsequent CVD events. Identification can be accomplished with a single blood test, as recommended by the ESC/EAS 2019 guidelines^100^ or through genotyping to identify variants at the *LPA* locus known to be associated with elevated Lp(a) levels. The utility of genetic data as a tool for predicting an individual’s future disease risk is an area of active research and debate.^127^^,^^128^ As genetic data, including *LPA* genotype, becomes more readily available to patients and clinicians, it may provide incremental information to supplement what can be offered from circulating Lp(a) measurement in the assessment of lifetime CVD risk. This genetic approach nonetheless needs close evaluation before being employed in clinical practice in order to ensure the most informative genetic variants are used, their impact on Lp(a) concentration and isoform size is well understood and such a test is applicable to all ancestral groups.^129^ In the nearer term, genetic information could prove valuable for evaluating pharmacogenetic interactions of RNAi therapeutics, and for identifying likely responder patients for inclusion in clinical trials.

## 10. Conclusion

Lp(a) is a well-identified risk factor for a range of CVDs. It has a potent causal effect on the onset and development of atherosclerotic, atherothrombotic, myocardial, and valvular disease, yet no specific and potent means of reducing Lp(a) is currently available for patients. Existing lipid-modifying therapies exert modest and variable effects on circulating Lp(a), while behavioural and dietary interventions^130^ are ineffective. RNAi therapeutics have considerable promise for treatment of long-term conditions with at least three experimental medicines for reducing circulating Lp(a). Preclinical and early clinical studies suggest that these emerging drugs may provide effective management of Lp(a)-mediated disease in a safe and acceptable manner. Although Lp(a) has previously been under-recognized as a CV risk factors, the work of investigators and of patient advocacy groups, such as the Lipoprotein(a) Association (www.familylipoproteina.org), the FH Foundation (www.thefhfounation.org), Heart UK (www.heartuk.org.uk), and FH Europe (www.fheurope.org) is ensuring that patients with raised Lp(a) are aware of their risk status and potential treatment options. If the novel Lp(a)-lowering agents prove safe and effective, they hold the potential to have an important beneficial impact on the health of patients, their families and of the wider population.
